# Cucurbitacin B Exerts Significant Antidepressant-Like Effects in a Chronic Unpredictable Mild Stress Model of Depression: Involvement of the Hippocampal BDNF-TrkB System

**DOI:** 10.1093/ijnp/pyad052

**Published:** 2023-08-21

**Authors:** Jian-Bin Ge, Bo Jiang, Tian-Shun Shi, Wei-Yu Li, Wei-Jia Chen, Bao-Lun Zhu, Zheng-Hong Qin

**Affiliations:** Department of Pharmacology and Laboratory of Aging and Nervous Disease, Jiangsu Key Laboratory of Neuropsychiatric Diseases, College of Pharmaceutical Science, Soochow University, Suzhou, Jiangsu, China; Department of Pharmacy, Affiliated Nantong Rehabilitation Hospital of Nantong University, Nantong, Jiangsu, China; Department of Pharmacology, School of Pharmacy, Nantong University, Nantong, Jiangsu, China; Department of Pharmacology, School of Pharmacy, Nantong University, Nantong, Jiangsu, China; Department of Pharmacology, School of Pharmacy, Nantong University, Nantong, Jiangsu, China; Department of Pharmacology, School of Pharmacy, Nantong University, Nantong, Jiangsu, China; Department of Pharmacology, School of Pharmacy, Nantong University, Nantong, Jiangsu, China; Department of Pharmacology and Laboratory of Aging and Nervous Disease, Jiangsu Key Laboratory of Neuropsychiatric Diseases, College of Pharmaceutical Science, Soochow University, Suzhou, Jiangsu, China

**Keywords:** Brain-derived neurotrophic factor, cucurbitacin B, depression, hippocampus, tyrosine kinase B

## Abstract

**Background:**

Although depression has been a serious neuropsychiatric disorder worldwide, current antidepressants used in clinical practice have various weaknesses, including delayed onset and low rates of efficacy. Recently, the development of new antidepressants from natural herbal medicine has become one of the important research hotspots. Cucurbitacin B is a natural compound widely distributed in the Cucurbitaceae and Cruciferae families and has many pharmacological activities. The present study aimed to investigate whether cucurbitacin B possess antidepressant-like effects in mice.

**Methods:**

The antidepressant-like effects of cucurbitacin B on mice behaviors were explored using the forced swim test, tail suspension test, open field test, sucrose preference test, and a chronic unpredictable mild stress model of depression together. Then, western blotting and immunofluorescence were used to examine the effects of cucurbitacin B on the brain-derived neurotrophic factor (BDNF)-tyrosine kinase B (TrkB) signaling cascade and neurogenesis in the hippocampus of mice. Furthermore, BDNF-short hairpin RNA, K252a, and p-chlorophenylalanine methyl ester were adopted together to determine the antidepressant mechanism of cucurbitacin B.

**Results:**

It was found that administration of cucurbitacin B indeed produced notable antidepressant-like effects in mice, which were accompanied with significant promotion in both the hippocampal BDNF-TrkB pathway and neurogenesis. The antidepressant mechanism of cucurbitacin B involves the hippocampal BDNF-TrkB system but not the serotonin system.

**Conclusions:**

Cucurbitacin B has the potential to be a novel antidepressant candidate.

**Significance Statement**

In recent years, the development of new antidepressants from natural herbal medicine has become one of the important research hotspots. As a widely distributed natural compound, cucurbitacin B has many pharmacological actions, such as anti-inflammatory, antitumor, and neuroprotective effects. To our knowledge, the present study is the first comprehensive evidence showing that cucurbitacin B also possesses antidepressant-like efficacy that involves the hippocampal BDNF signaling and neurogenesis; it not only extends the knowledge of its pharmacological activities but also provides a novel antidepressant candidate.

## INTRODUCTION

As one of the most common psychiatric disorders in the world, depression is characterized by dysregulation of emotion and mood as well as abnormalities of cognitive function, sleep, appetite, and metabolism (Cui, 2015). Moreover, depression is known to be a leading cause of disability worldwide and causes huge economic burden to both society and families (Yang et al., 2015). However, the currently available antidepressants used in clinical practice (selective serotonin reuptake inhibitors, serotonin and norepinephrine reuptake inhibitors, monamine oxidase inhibitors, etc.) have important limitations such as delayed onset from weeks to months and low rates of efficacy (Alamo and López-Muñoz, 2009; Blier and El Mansari, 2013). Therefore, it is still necessary to conduct further research and development of novel and more effective antidepressants.

In the 20th century, dysfunction of the monoaminergic system was considered to be the cause of depression, and so, selective serotonin reuptake inhibitors and serotonin and norepinephrine reuptake inhibitors are the most widely used antidepressants (López-Muñoz and Alamo, 2009; Pereira and Hiroaki-Sato, 2018). In the 21st century, another popular hypothesis of depression suggests that neurotrophic factors (especially brain-derived neurotrophic factor [BDNF]) and adult neurogenesis are closely implicated in not only the pathophysiology of depression but also the behavioral responses to antidepressants (Adlam and Zaman, 2013; Micheli et al., 2018; Castrén and Monteggia, 2021). It is well-known that BDNF activates the function of cAMP response element-binding protein (CREB) in the nucleus by combining tyrosine kinase B (TrkB) receptor on the cell membrane and then promoting the downstream mitogen-activated protein kinase-extracellular regulated protein kinase (ERK) and phosphoinositide 3-kinase-protein kinase B (AKT) signaling pathways (Sasi et al., 2017). It is also known that depression is accompanied with decreased expression of BDNF and phosphorylated CREB (pCREB) in the hippocampus, whereas chronic antidepressant treatments reverse these molecular changes (Blendy, 2006; Kozisek et al., 2008). Moreover, adult neurogenesis in the dentate gyrus (DG) in the hippocampus is regulated by BDNF and chronic stress and required for the therapeutic responses of many antidepressants (Mahar et al., 2014; Micheli, et al., 2018). Therefore, stimulation of the BDNF signaling pathway and hippocampal neurogenesis could provide a novel approach to the treatment of depression.

Currently, the use of complementary and alternative medicine among psychiatric disorders, especially depression and anxiety, is a common phenomenon. Herbal medicine is one of the most commonly used forms of complementary and alternative medicine therapies (Liu et al., 2015). Among herbal medicine worldwide, traditional Chinese medicines are great sources of compounds with medical uses. To date, numerous compounds in traditional Chinese medicines have been reported to possess antidepressant-like efficacy in rodent models of depression, such as ginsenoside Rg1, ginsenoside Rg2, andrographolide, oroxylin A, tetrahydroxystilbene glucoside, tetramethylpyrazine, and so on (Jiang et al., 2012, 2015; Ren et al., 2017; Wang et al., 2017; Zhang et al., 2019; Wu et al., 2022).

Cucurbitacin B is a natural compound widely distributed and has many derivatives. This compound is mainly found in plants in the Cucurbitaceae and Cruciferae families, including *Cucumis Melo*, *Cucurbita Andreana*, *Ecballium Elaterium*, *Wilbrandia Ebracteata*, and *Trichosanthes Cucumerina* (Dai et al., 2023). Cucurbitacin B has been well-demonstrated to possess a variety of pharmacological activities such as anti-inflammatory, antioxidant, antiviral, hypoglycemic, hepatoprotective, neuroprotective, and anticancer effects (Dai et al., 2023). These pharmacological activities largely contribute to the prevention and treatment of cucurbitacin B against various diseases such as inflammatory diseases, neurodegenerative diseases, diabetes mellitus, and cancers (Dai et al., 2023). Recently, it has been reported that administration of cucurbitacin B significantly promoted hippocampal neurogenesis in ICR and APP/PS1 mice and rescued working memory in APP/PS1 mice (Li et al., 2019). Moreover, cucurbitacin B treatment significantly induced the phosphorylation of ERK and CREB (Li et al., 2019), 2 key downstream molecules of BDNF, in PC12 cells. According to these studies, we speculated here that cucurbitacin B may have antidepressant-like effects, and various methods were used in this study to investigate this assumption.

## MATERIALS AND METHODS

### Animals

All procedures involving mice were conducted according to the ARRIVE guidelines (Kilkenny et al., 2010; McGrath and Lilley, 2015) and approved by the Animal Welfare Committees of Soochow University and Nantong University. SLAC Laboratory Animal Co., Ltd. (Shanghai, China) provided C57BL/6J mice (male, 8 weeks) for this study. Before use, all mice were housed (5 per cage, M2 type) under standard conditions (12-hour-light/-dark cycle, lights on from 7:00 am to 7:00 pm; 23°C ± 1°C ambient temperature; 55% ± 10% relative humidity; noise less than 50 dB; ammonia concentration less than 14 mg/m^3^; 24 hours of air circulation; and bedding replacement twice a week) for 1 week with ad libitum access to water and rodent chow. The mice were subjected to stratified randomization according to their body weights. All behavioral experiments were performed in the daytime (8:00 am–5:00 pm). To perform in vitro studies, the mice were killed at 9:00 am; the mice were anaesthetized using carbon dioxide and then killed by cervical dislocation.

### Materials

Cucurbitacin B (purity >99%), fluoxetine, K252a, and p-chlorophenylalanine methyl ester (PCPA) were purchased from Target Mol (Boston, MA, United States). Adeno-associated virus (AAV)-BDNF-short hairpin RNA (shRNA)-enhanced green fluorescent protein and AAV-Control-shRNA-enhanced green fluorescent protein were provided by GeneChem Co., Ltd (Shanghai, China). The doses of cucurbitacin B (2.5, 5, 10, and 20 mg/kg), fluoxetine (20 mg/kg), K252a (25 μg/kg), and PCPA (300 mg/kg) were chosen according to previous studies (Jiang et al., 2012; Ren et al., 2017; Wang et al., 2017; Sallam et al., 2018; Xue et al., 2021). The vehicle for cucurbitacin B, fluoxetine, K252a, and PCPA was normal saline containing 10% dimethyl sulfoxide and 20% Cremaphor EL. All drugs were i.p. injected at a concentration of 10 mL/kg. AAV-BDNF-shRNA or AAV-Control-shRNA were bilaterally injected into the hippocampus of C57BL/6J mice.

### Forced Swimming Test (FST)

This is a widely used test for assessing potential antidepressant-like medications. In the present study, the FST was performed according to Porsolt et al. with slight modifications (Porsolt et al., 1977). In brief, each test mouse was placed into a cylindrical container filled with water (20 cm in diameter, 45 cm high, 15 cm water depth) maintained at 25°C. The immobility time was recorded during the last 4 minutes of the total 6-minute swimming (immobility means that mice stop swimming and make no active movements). Afterwards, each mouse was dried and returned to its home cage. The observer was unaware of animal grouping.

Additional materials, methods, and two supplementary figures are included in supplementary information.

### Statistical Analyses

All data are presented as means ± SEM. For statistical analyses, SPSS 26.0 software (SPSS Inc, Chicago, IL, USA) was used to perform 1-way ANONA + Tukey test or 2-way ANONA + Bonferroni test. A value of *P* < .05 was considered statistically significant.

## RESULTS

### Single Administration of Cucurbitacin B Displays Antidepressant-Like Potential in FST and Tail Suspension Test (TST)

**Figure 1.**
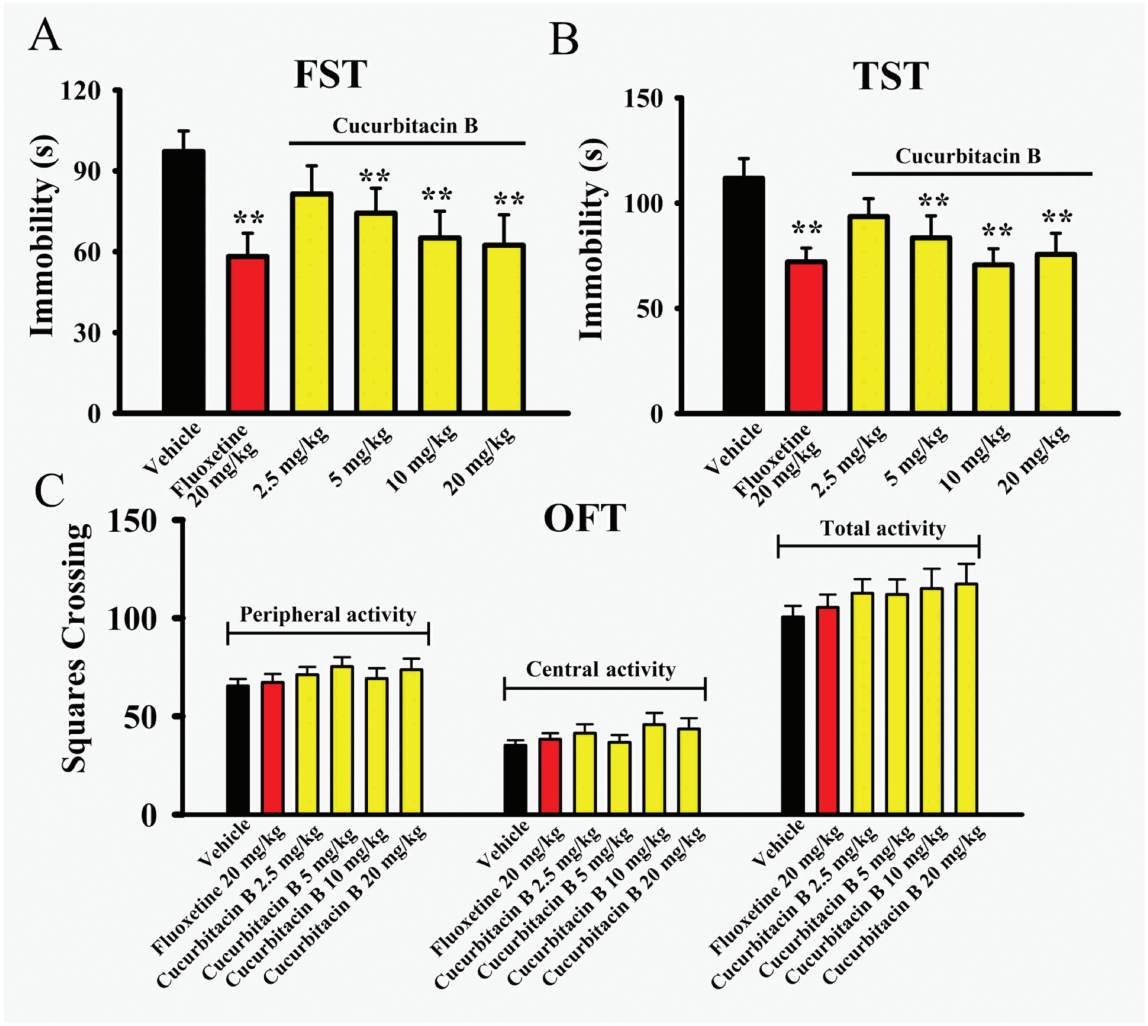
Single administration of cucurbitacin B displays antidepressant-like potential in the FST and TST. Naïve C57BL/6J mice were subjected to a single i.p. injection of vehicle, fluoxetine (20 mg/kg), or cucurbitacin B (2.5, 5, 10, and 20 mg/kg), followed by the FST, TST, or OFT (after 30 minutes). (A and B) Treatment of cucurbitacin B (5, 10, and 20 mg/kg) and fluoxetine significantly decreased the immobility time of naive mice in both the FST and TST. (C) Neither cucurbitacin B nor fluoxetine produced significant influence on the locomotor activity of naive mice in the OFT. All results are expressed as means ± SEM (n = 10); ^**^P < .01 vs vehicle. For statistical analyses, 1-way ANOVA and Tukey test were used together. ANOVA, analysis of variance; FST, forced swimming test; OFT, open field test; SEM, standard error of mean; TST, tail suspension test.

As to whether cucurbitacin B has antidepressant potential, both the FST and TST were adopted. Naïve mice were subjected to a single i.p. injection of vehicle, fluoxetine, or cucurbitacin B, followed by the FST, TST, or open field test (OFT). Figure 1A illustrates the FST data and shows that the immobility duration of mice in the vehicle-treated control group was significantly longer than that of mice in the fluoxetine-treated and cucurbitacin B-treated groups. Detailed analyses reveal that compared with the vehicle, 5-, 10-, and 20-mg/kg treatment of cucurbitacin B induced a 23.6% ± 4.21%, 33.1% ± 5.15%, and 35.8% ± 4.46% decrease in mouse immobility in the FST, respectively (n = 10, *P < *.01 vs vehicle). Administration of 20 mg/kg fluoxetine (the positive control) also significantly reduced mouse immobility in the FST (n = 10, *P < *.01 vs vehicle), as expected, whereas treatment of 2.5 mg/kg cucurbitacin B induced nonsignificant effects (n = 10). Moreover, the effects of 5 mg/kg cucurbitacin B were comparable with those of 10 mg/kg cucurbitacin B. One-way ANOVA analyses indicated a notable effect of drug treatment (F_(5,54)_ = 23.746, *P < *.01). Figure 1B illustrates the TST data and shows that compared with the vehicle, administration of 5, 10, and 20 mg/kg cucurbitacin B induced a 25.3% ± 4.17%, 36.8% ± 6.24%, and 32.4% ± 5.79% reduction in mouse immobility in the TST, respectively (n = 10, *P < *.01 vs vehicle). The effects of 5 mg/kg cucurbitacin B were nearly the same as those of 10 mg/kg cucurbitacin B and 20 mg/kg fluoxetine. One-way ANOVA analyses also indicated a notable effect of drug treatment (F_(5,54)_ = 18.582, *P < *.01). Therefore, 5 and 10 mg/kg were chosen as the doses of cucurbitacin B in the following studies.

Moreover, the OFT results in Figure 1C reveal no significant effects of cucurbitacin B on mice locomotor activity. No significant differences were found among all groups in the number of squares that a mouse crossed in the central or peripheral area (n = 10). Correspondingly, 1-way ANOVA analyses showed no effects of drug treatments (F_(5,66)_ = 2.026, *P* = .143). Collectively, cucurbitacin B possesses antidepressant potential in mice.

### Repeated Cucurbitacin B Treatment Prevented Chronic Unpredictable Mild Stress (CUMS)-Induced Depressive-Like Behaviors

**Figure 2.**
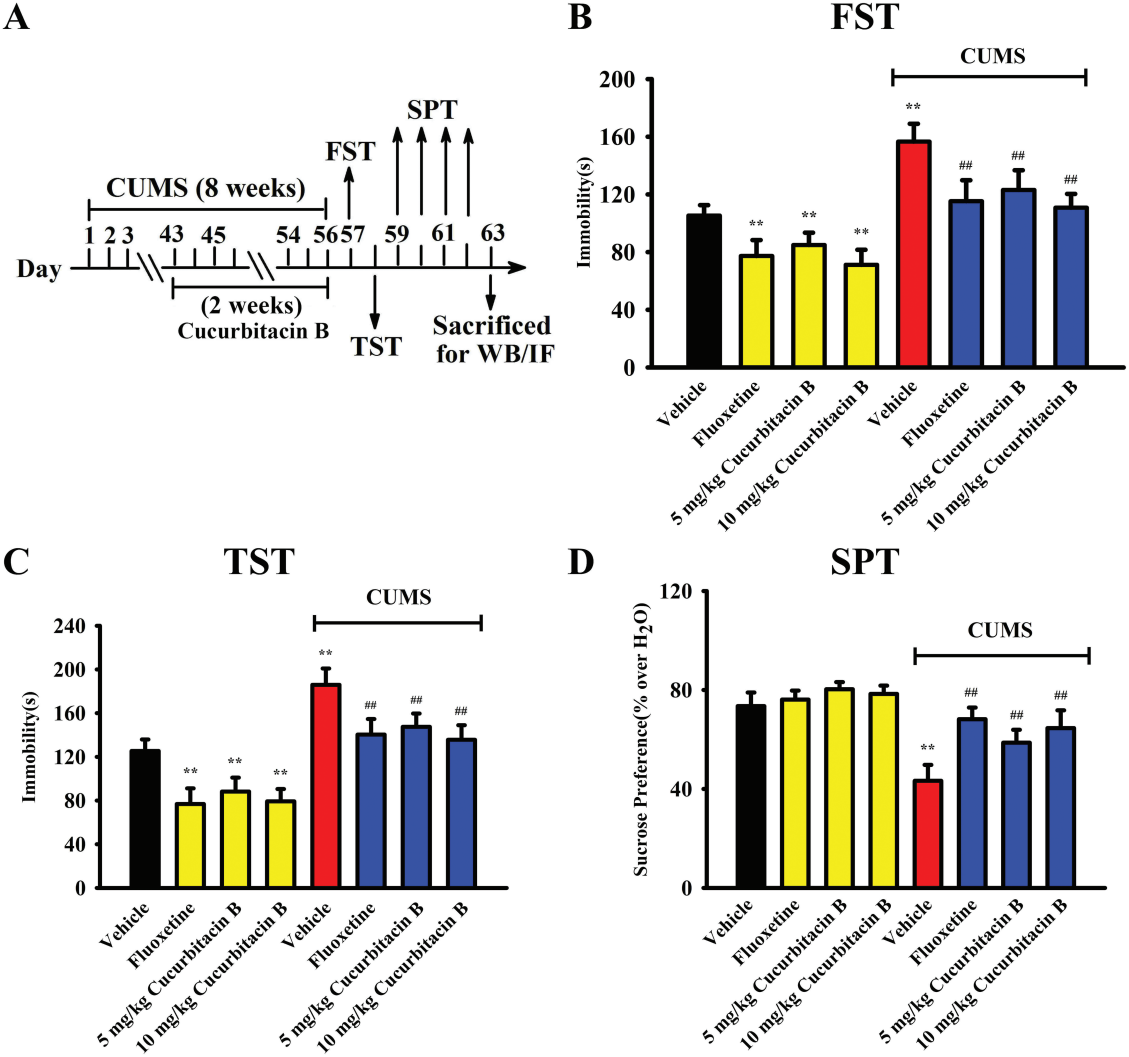
Repeated administration of cucurbitacin B fully prevented the CUMS-induced depressive-like behaviors in mice. (A) A schematic diagram that describes the timeline of the experimental procedures. Repeated i.p. injection of vehicle, fluoxetine (20 mg/kg), or cucurbitacin B (5 and 20 mg/kg) were given to the CUMS-exposed mice (daily, during the last 2 weeks), followed by the FST, TST, and SPT. (B and C) Mice in the (CUMS + cucurbitacin B)-treated and (CUMS + fluoxetine)-treated groups spent significantly less time being immobile in both the FST and TST than mice in the (CUMS + vehicle)-treated group. (D) Mice in the (CUMS + cucurbitacin B)-treated and (CUMS + fluoxetine)-treated groups displayed notably higher level of sucrose preference in the SPT than mice in the (CUMS + vehicle)-treated group. All results are expressed as means ± SEM (n = 10); ^**^P < .01 vs vehicle; ^##^*P < *.01 vs (CUMS + vehicle). For statistical analyses, 2-way ANOVA and Bonferroni test were used together. ANOVA, analysis of variance; CUMS, chronic unpredictable mild stress; FST, forced swimming test; OFT, open field test; SEM, standard error of mean; TST, tail suspension test.

The CUMS model of depression was established, and administration of cucurbitacin B was performed daily during the last 2 weeks (Figure 2A). As shown in Figure 2B and C, mice in the (CUMS + vehicle)-treated group exhibited significantly more immobility in the FST and TST than mice in the vehicle-treated control group (n = 10, *P < *.01 vs vehicle), whereas repeated administration of both fluoxetine and cucurbitacin B significantly antagonized the enhancing effects of CUMS on mice immobility in the FST and TST (n = 10, *P < *.01 vs CUMS + vehicle). Detailed data analyses indicate that exposure of CUMS increased the immobility of mice in the FST and TST by 48.7% ± 6.06% and 48.2% ± 7.23%, respectively. In contrast, the FST immobility of mice in the (CUMS + vehicle)-treated group was decreased by 21.4% ± 5.26% and 29.2% ± 4.73% under administration of 5 and 10 mg/kg cucurbitacin B, respectively. The TST immobility of mice in the (CUMS + vehicle)-treated group was decreased by 20.7% ± 3.48% and 27.1% ± 5.14% under administration of 5 and 10 mg/kg cucurbitacin B, respectively. The sucrose preference test (SPT) results are displayed in Figure 2C. CUMS exposure induced to a 41.1% ± 7.54% decrease in the sucrose preference of mice compared with that of the vehicle-treated control mice (n = 10, *P < *.01 vs vehicle), and this behavioral change was fully reversed by treatment of fluoxetine and cucurbitacin B (n = 10, *P < *.01 vs CUMS + vehicle). Detailed data analyses indicated that the sucrose preference of mice in the (CUMS + vehicle)-treated group increased by 35.6% ± 6.09% and 49.2% ± 8.23% under treatment of 5 and 10 mg/kg cucurbitacin B, respectively. Taken together, cucurbitacin B indeed has antidepressant-like efficacy in mice.

For the FST results, 2-way ANOVA analyses revealed a significant interaction (F_(3,72)_ = 16.235, *P < *.01) with significant effects for CUMS (F_(1,72)_ = 25.429, *P < *.01) and drug treatment (F_(3,72)_ = 21.664, *P < *.01). For the TST results, 2-way ANOVA analyses revealed a significant interaction (F_(3,72)_ = 14.251, *P < *.01) with significant effects for CUMS (F_(1,72)_ = 27.189, *P < *.01) and drug treatment (F_(3,72)_ = 23.024, *P < *.01). For the SPT results, 2-way ANOVA analyses also revealed a significant interaction (F_(3,72)_ = 13.284, *P < *.01) with significant effects for CUMS (F_(1,72)_ = 26.338, *P < *.01) and drug treatment (F_(3,72)_ = 20.307, *P < *.01).

### Repeated Cucurbitacin B Treatment Antagonized Downregulating Effects of CUMS on Hippocampal BDNF System

**Figure 3.**
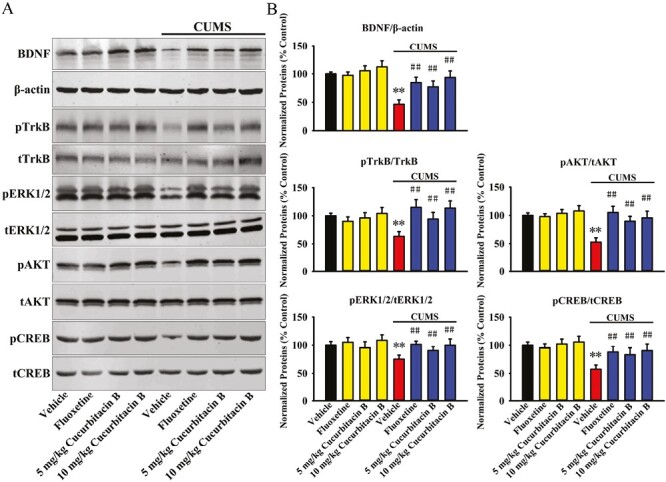
Repeated administration of cucurbitacin B notably antagonized the downregulating effects of CUMS on the hippocampal BDNF signaling pathway in mice. (A) Representative western blotting images show the effects of CUMS, fluoxetine, and cucurbitacin B on the protein expression of BDNF, β-actin, pTrkB, tTrkB, pERK1/2, tERK1/2, pAKT, tAKT, pCREB, and tCREB in the hippocampus of mice. (B) Corresponding band analyses reveal that mice in the (CUMS + cucurbitacin B)-treated and (CUMS + fluoxetine)-treated groups had evidently higher protein levels of hippocampal BDNF, pTrkB, pERK1/2, pAKT, and pCREB than mice in the (CUMS + vehicle)-treated group. In contrast, the protein levels of tTrkB, tERK1/2, tAKT, tCREB, and β-actin remain constant between all groups. All results are expressed as means ± SEM (n = 5); ^**^P < .01 vs vehicle; ^##^*P < *.01 vs (CUMS + vehicle). For statistical analyses, 2-way ANOVA and Bonferroni’s test were used together. ANOVA, analysis of variance; BDNF, brain-derived neurotrophic factor; CUMS, chronic unpredictable mild stress; pTrkB, protein phosphorylation of tyrosine kinase B; SEM, standard error of mean; tTrkB, tyrosine kinase B.

Afterwards, western blotting was adopted to examine the expression of the hippocampal BDNF signaling cascade following CUMS and drug treatments, and the results are summarized in Figure 3A and B. It was found that the protein expression of hippocampal BDNF was significantly decreased in mice in the (CUMS + vehicle)-treated group compared with that in the vehicle-treated group (n = 5, *P < *.01 vs vehicle), whereas administration of 5 and 10 mg/kg cucurbitacin B increased it by 66.4% ± 9.55% and 101.5% ± 14.12%, respectively (n = 5, *P < *.01 vs CUMS + vehicle). We also examined the protein phosphorylation of TrkB (pTrkB), ERK1/2 (pERK1/2), AKT (pAKT), and CREB (pCREB), the downstream signaling molecules of BDNF. In parallel with BDNF, the protein levels of hippocampal pTrkB, pERK1/2, pAKT, and pCREB were all significantly decreased in mice in the (CUMS + vehicle)-treated group compared with that in the vehicle-treated group (n = 5, *P < *.01 vs vehicle), whereas administration of 5 and 10 mg/kg cucurbitacin B fully reversed these molecular changes (n = 5, *P < *.01 vs CUMS + vehicle). In contrast, the protein levels of total TrkB (tTrkB), ERK1/2 (tERK1/2), AKT (tAKT), CREB (tCREB), and β-actin were unchanged among all groups. Taken together, the antidepressant-like effects of cucurbitacin B in mice may involve the hippocampal BDNF system.

For the BDNF data, 2-way ANOVA analyses showed a significant interaction (F_(3,32)_ = 20.117, *P < *.01) with significant effects for CUMS (F_(1,32)_ = 32.124, *P < *.01) and drug treatment (F_(3,32)_ = 25.478, *P < *.01). For the pTrkB data, 2-way ANOVA analyses showed a significant interaction (F_(3,32)_ = 15.471, *P < *.01) with significant effects for CUMS (F_(1,32)_ = 19.705, *P < *.01) and drug treatment (F_(3,32)_ = 23.387, *P < *.01). For the pERK1/2 data, 2-way ANOVA analyses showed a significant interaction (F_(3,32)_ = 11.213, *P < *.01) with significant effects for CUMS (F_(1,32)_ = 17.626, *P < *.01) and drug treatment (F_(3,32)_ = 14.305, *P < *.01). For the pAKT data, 2-way ANOVA analyses showed a significant interaction (F_(3,32)_ = 21.135, *P < *.01) with significant effects for CUMS (F_(1,32)_ = 30.138, *P < *.01) and drug treatment (F_(3,32)_ = 25.806, *P < *.01). For the pCREB data, 2-way ANOVA analyses also showed a significant interaction (F_(3,32)_ = 18.187, *P < *.01) with significant effects for CUMS (F_(1,32)_ = 27.293, *P < *.01) and drug treatment (F_(3,32)_ = 23.054, *P < *.01).

### Repeated Cucurbitacin B Treatment Reversed Downregulating Effects of CUMS on Hippocampal Neurogenesis

**Figure 4.**
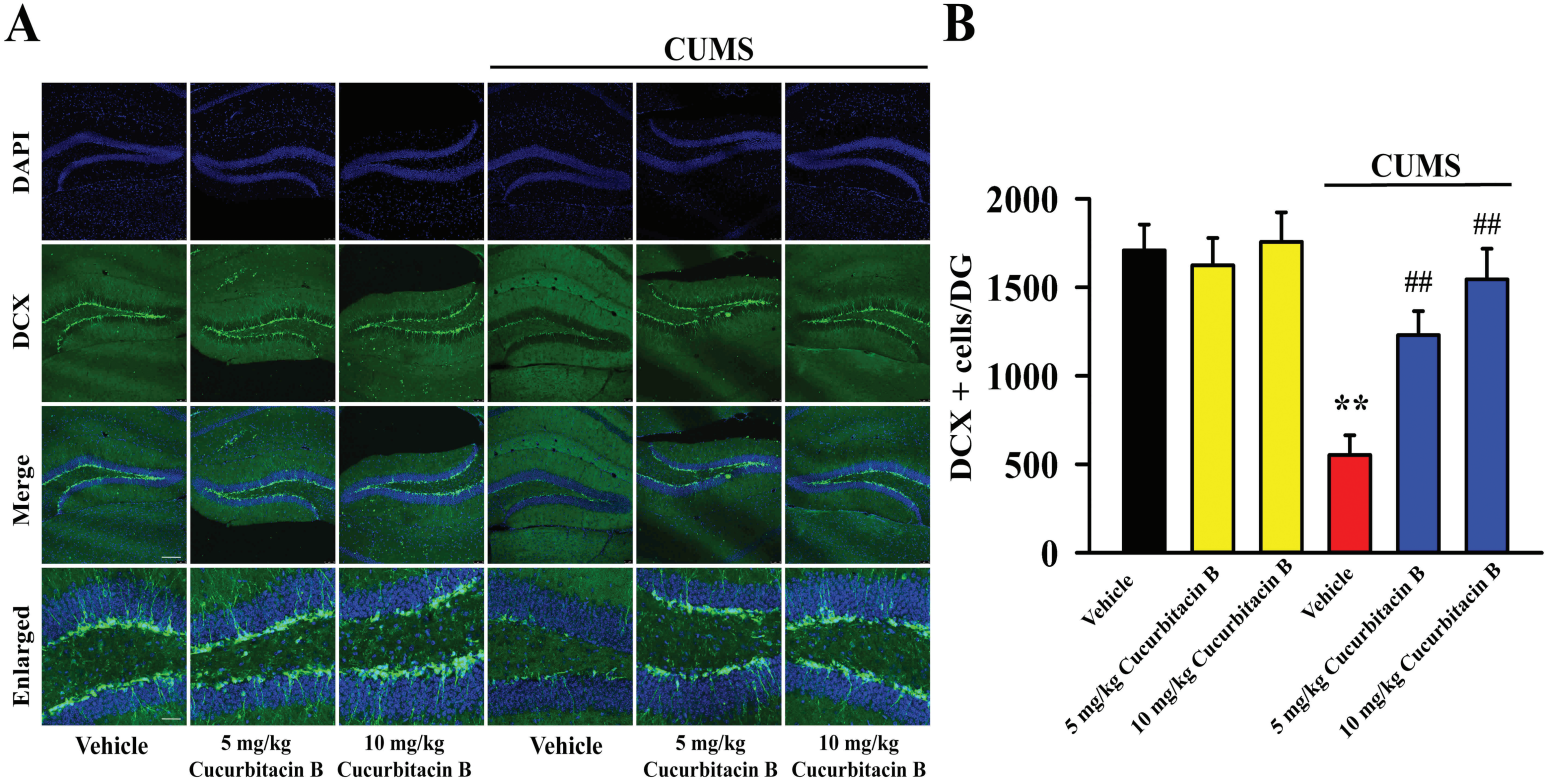
Repeated administration of cucurbitacin B significantly reversed the downregulating effects of CUMS on hippocampal neurogenesis in mice. (A) Representative immunofluorescence images show the effects of CUMS, fluoxetine, and cucurbitacin B on the DCX^+^ cells amount in the DG region of mice. The scale bar for representative images is 150 μm. The scale bar for enlarged images is 37.5 μm. (B) Corresponding image analyses reveal that mice in the (CUMS + cucurbitacin B)-treated and (CUMS + fluoxetine)-treated groups exhibited evidently more DCX^+^ cells in the DG region than mice in the (CUMS + vehicle)-treated group. All results are expressed as means ± SEM (n = 5); ^**^P < .01 vs vehicle; ^##^*P < *.01 vs (CUMS + vehicle). For statistical analyses, 2-way ANOVA and Bonferroni test were used together. ANOVA, analysis of variance; CUMS, chronic unpredictable mild stress; DCX, doublecortin; SEM, standard error of mean.

Doublecortin (DCX) immunofluorescence in the DG region was performed to examine whether cucurbitacin B administration reversed the downregulating effects of CUMS on hippocampal neurogenesis in mice. As shown in Figure 4A and B, compared with the vehicle-treated control group, exposure to CUMS led to a 67.7% ± 10.35% reduction in the amount of DCX^+^ cells in the DG of mice (n = 5, *P < *.01 vs vehicle). In contrast, the amount of DCX^+^ cells in the DG of mice in the (CUMS + vehicle)-treated group increased by 122.8% ± 15.81% and 180.1% ± 21.35% under administration of 5 and 10 mg/kg cucurbitacin B, respectively (n = 5, *P < *.01 vs CUMS + vehicle). Two-way ANOVA analyses showed a significant interaction (F_(3,32)_ = 28.603, *P < *.01) with significant effects for CUMS (F_(1,32)_ = 42.058, *P < *.01) and drug treatment (F_(3,32)_ = 36.147, *P < *.01). Therefore, cucurbitacin B has beneficial effects against the CUMS-induced dysfunction in the hippocampal neurogenesis.

### Blockade of Hippocampal BDNF System Abolished Antidepressant-Like Effects of Cucurbitacin B

**Figure 5.**
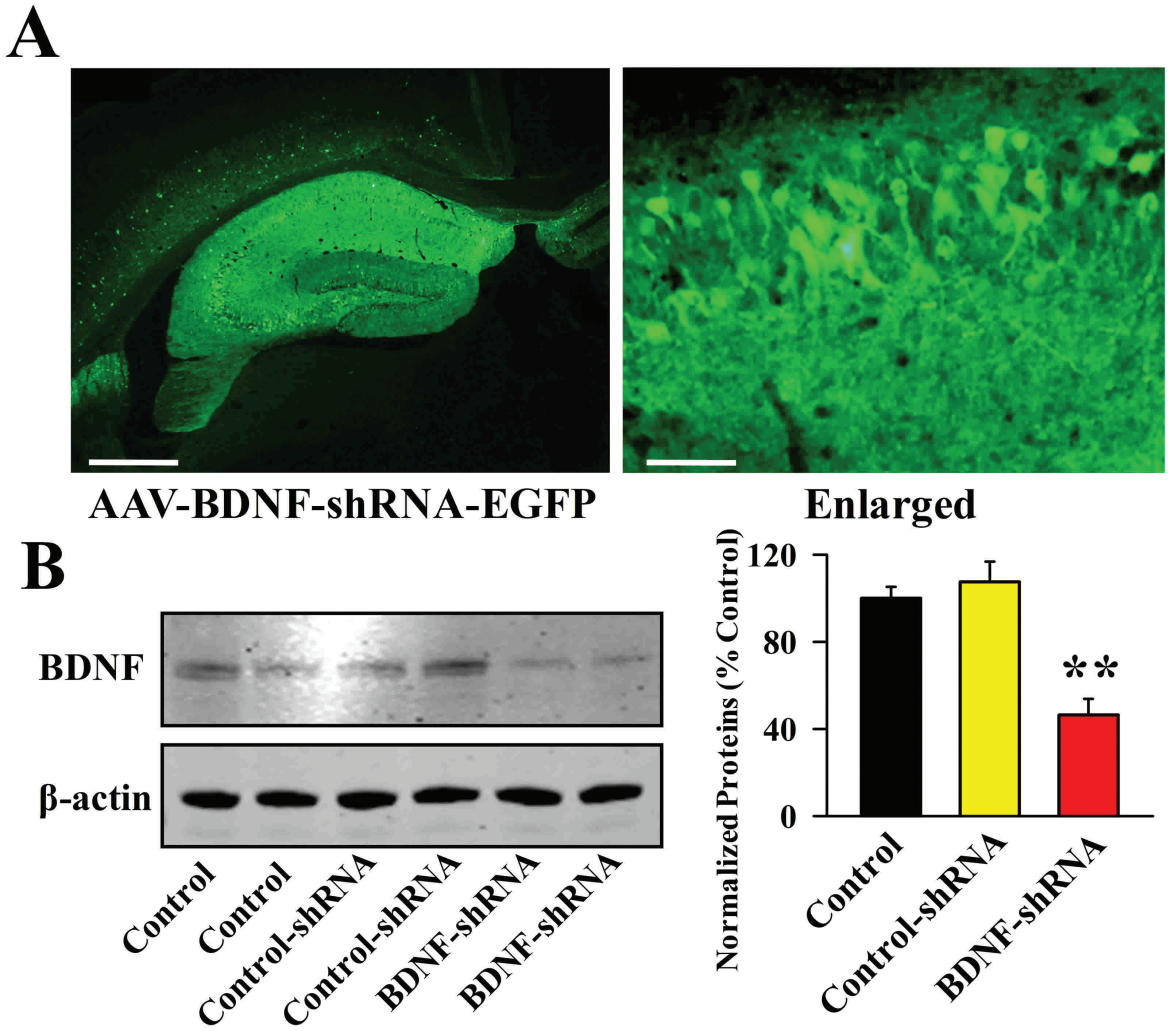
The usage of BDNF-shRNA to successfully knock down the expression of TrkB in the hippocampus of mice. (A) Representative fluorescence images of a fixed hippocampal slice expressing AAV-BDNF-shRNA-EGFP. The scale bar for representative image is 400 μm. The scale bar for enlarged image is 50 μm. (B) Corresponding western blotting detection confirmed the silencing effects of BDNF-shRNA on the protein expression of hippocampal BDNF in mice. All results are expressed as means ± SEM (n = 5); ^**^P < .01 vs vehicle. For statistical analyses, 1-way ANOVA and Tukey test were used together. ANOVA, analysis of variance; BDNF, brain-derived neurotrophic factor; CUMS, chronic unpredictable mild stress; SEM, standard error of mean; shRNA, short hairpin RNA; TrkB, tyrosine kinase B.

**Figure 6.**
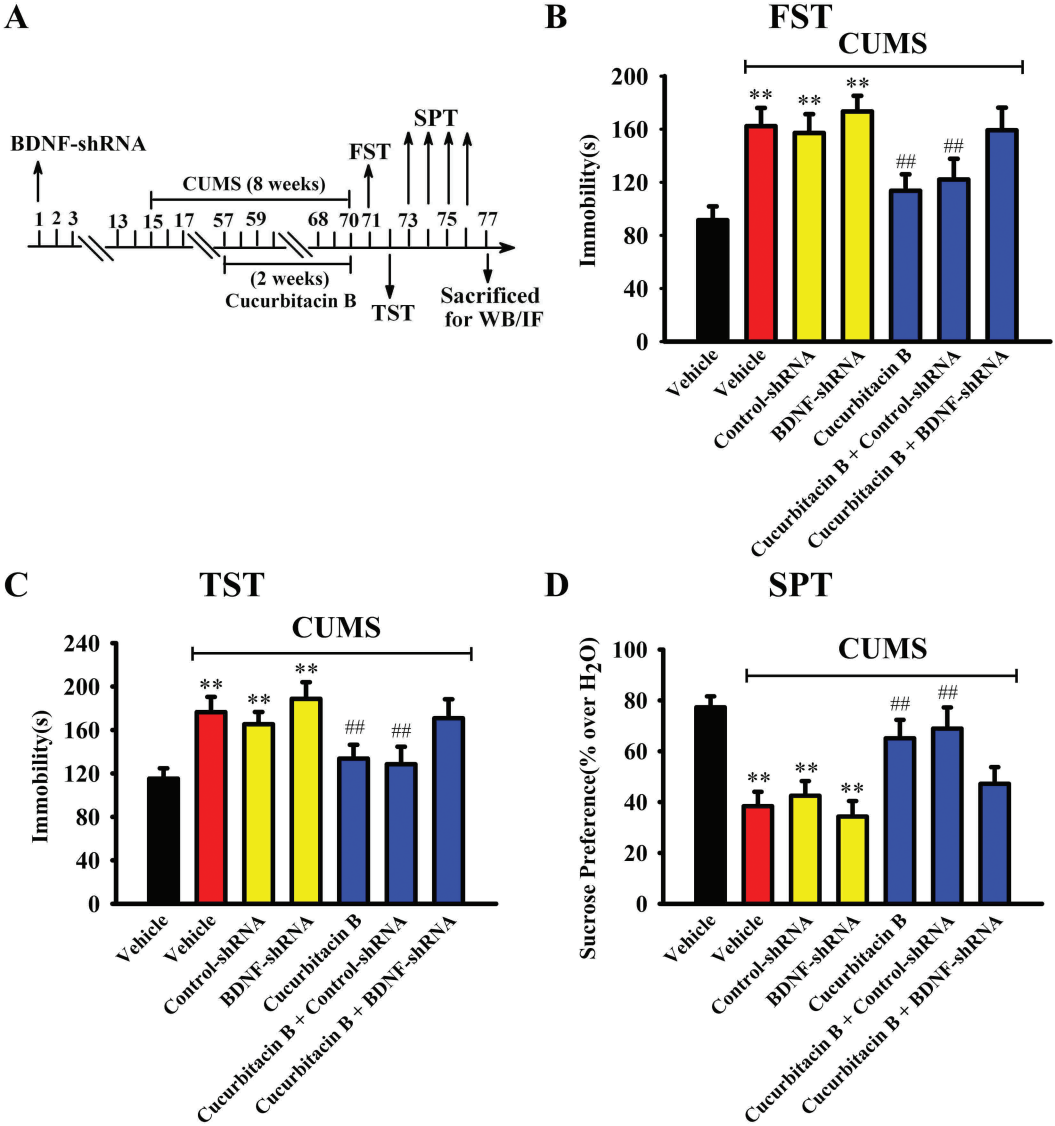
Genetic blockade of the hippocampal BDNF-TrkB system evidently abolished the antidepressant-like effects of cucurbitacin B on mice behaviors. (A) A schematic diagram describes the timeline of the experimental procedures. Mice pretreated with BDNF-shRNA were kept for 2 weeks and then exposed to CUMS (8 weeks) and cucurbitacin B administration (10 mg/kg; daily, during the last 2 weeks), followed by the FST, TST, and SPT. (B and C) Mice in the (CUMS + cucurbitacin B + BDNF-shRNA)-treated group spent significantly more time being immobile in both the FST and TST than mice in the (CUMS + cucurbitacin B)-treated and (CUMS + cucurbitacin B + control-shRNA)-treated groups. (D) Mice in the (CUMS + cucurbitacin B + BDNF-shRNA)-treated group displayed notably lower level of sucrose preference in the SPT than mice in the (CUMS + cucurbitacin B)-treated and (CUMS + cucurbitacin B + control-shRNA)-treated groups. All results are expressed as means ± SEM (n = 10); ^**^P < .01 vs vehicle; ^##^*P < *.01 vs (CUMS + vehicle). For statistical analyses, 1-way ANOVA and Tukey test were used together. ANOVA, analysis of variance; BDNF, brain-derived neurotrophic factor; CUMS, chronic unpredictable mild stress; FST, forced swimming test; OFT, open field test; SEM, standard error of mean; shRNA, short hairpin RNA; TrkB, tyrosine kinase B; TST, tail suspension test.

**Figure 7.**
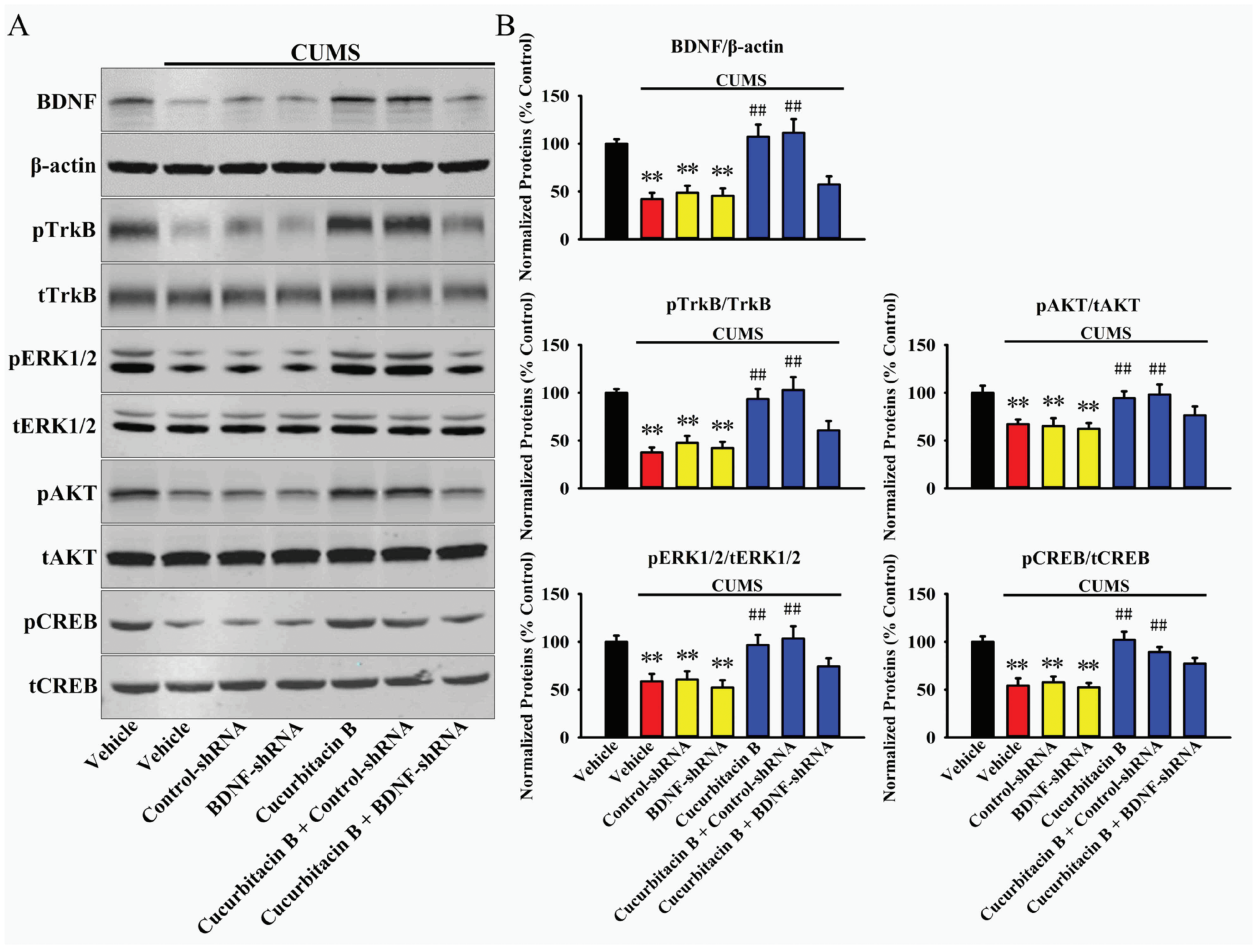
The usage of BDNF-shRNA evidently blocked the protecting effects of cucurbitacin B against the decreased hippocampal BDNF signaling in mice exposed to CUMS. (A) Representative western blotting images show the effects of CUMS, BDNF-shRNA, and cucurbitacin B on the protein expression of BDNF, β-actin, pTrkB, tTrkB, pERK1/2, tERK1/2, pAKT, tAKT, pCREB, and tCREB in the hippocampus of mice. (B) Corresponding band analyses reveal that mice in the (CUMS + cucurbitacin B + BDNF-shRNA)-treated group showed significantly lower protein levels of hippocampal BDNF, pTrkB, pERK1/2, pAKT, and pCREB than mice in the (CUMS + cucurbitacin B)-treated and (CUMS + cucurbitacin B + control-shRNA)-treated groups. In contrast, the protein levels of tTrkB, tERK1/2, tAKT, tCREB, and β-actin remain constant between all groups. All results are expressed as means ± SEM (n = 5); ^**^P < .01 vs vehicle; ^##^*P < *.01 vs (CUMS + vehicle). For statistical analyses, 1-way ANOVA and Tukey test were used together. ANOVA, analysis of variance; BDNF, brain-derived neurotrophic factor; CUMS, chronic unpredictable mild stress; SEM, standard error of mean; shRNA, short hairpin RNA; TrkB, tyrosine kinase B.

**Figure 8.**
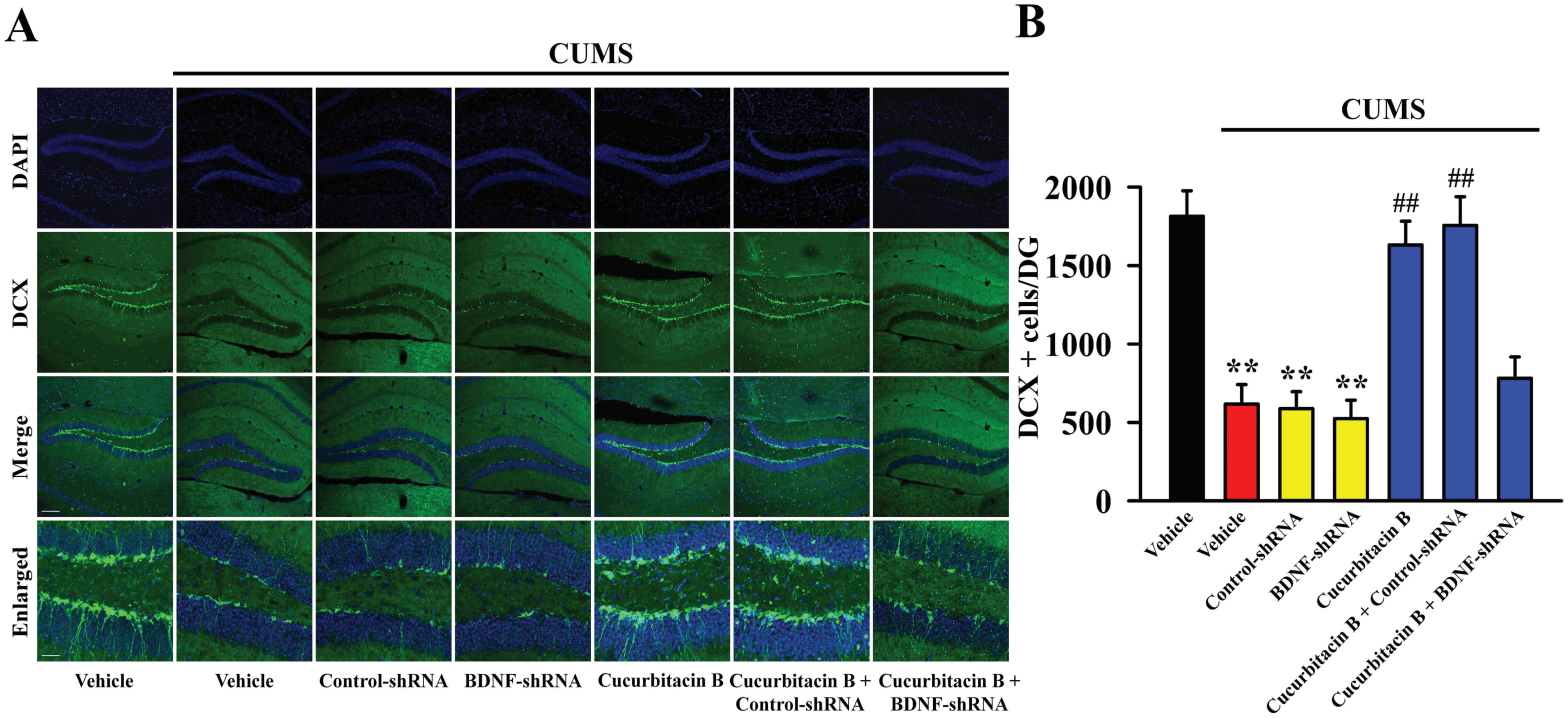
The usage of BDNF-shRNA significantly prevented the reversal effects of cucurbitacin B on the decreased hippocampal neurogenesis in mice subjected to CUMS. (A) Representative immunofluorescence images show the effects of CUMS, BDNF-shRNA, and cucurbitacin B on the amount of DCX^+^ cells in the DG region of mice. The scale bar for representative images is 150 μm. The scale bar for enlarged images is 37.5 μm. (B) Corresponding image analyses reveal that mice in the (CUMS + cucurbitacin B + BDNF-shRNA)-treated group exhibited notably less DCX^+^ cells in the DG region than mice in the (CUMS + cucurbitacin B)-treated and (CUMS + cucurbitacin B + control-shRNA)-treated groups. All results are expressed as means ± SEM (n = 5); ^**^P < .01 vs vehicle; ^##^*P < *.01 vs (CUMS + vehicle). For statistical analyses, 1-way ANOVA and Tukey test were used together. ANOVA, analysis of variance; BDNF, brain-derived neurotrophic factor; CUMS, chronic unpredictable mild stress; DCX, doublecortin; SEM, standard error of mean; shRNA, short hairpin RNA; TrkB, tyrosine kinase B.

From the above results it can be speculated that cucurbitacin B may produce antidepressant-like effects by promoting the hippocampal BDNF-TrkB system. To validate this assumption, BDNF-shRNA was used to selectively knock down the hippocampal expression of BDNF. As shown in Figure 5A and B, virus was stably expressed in the hippocampal neurons 2 weeks after stereotactic infusion, and the silencing efficacy of BDNF-shRNA was confirmed (1-way ANOVA: F_(2,12)_ = 16.276, *P < *.01, n = 5, *P < *.01 vs control). Here, mice infused with BDNF-shRNA were maintained for 2 weeks and then subjected to CUMS and 10-mg/kg administration of cucurbitacin B (Figure 6A). Afterwards, the behavioral tests were performed, and the results are summarized in Figure 6B–D. It was found that BDNF-shRNA infusion significantly abolished the decreasing effects of 10 mg/kg cucurbitacin B on FST immobility (1-way ANOVA: F_(6,63)_ = 23.923, *P < *.01) and TST immobility (1-way ANOVA: F_(6,63)_ = 19.778, *P < *.01) of mice subjected to CUMS (n = 10). Additionally, BDNF-shRNA infusion notably abolished the increasing effects of 10 mg/kg cucurbitacin B on the sucrose preference of mice subjected to CUMS (1-way ANOVA: F_(6,63)_ = 25.104, *P < *.01, n = 10). Next, western blotting was performed, and the results are displayed in Figure 7A and B. It was found that the usage of BDNF-shRNA significantly prevented the enhancing effects of 10 mg/kg cucurbitacin B on the expression of hippocampal BDNF (1-way ANOVA: F_(6,28)_ = 29.795, *P < *.01), pTrkB (1-way ANOVA: F_(6,28)_ = 33.667, *P < *.01), pERK1/2 (1-way ANOVA: F_(6,28)_ = 22.445, *P < *.01), pAKT (1-way ANOVA: F_(6,28)_ = 17.115, *P < *.01), and pCREB (1-way ANOVA: F_(6,28)_ = 24.659, *P < *.01) in mice subjected to CUMS (n = 5). The expression of tTrkB, tERK1/2, tAKT, tCREB, and β-actin was unchanged among all groups. Moreover, immunofluorescence was also performed, and the results are shown in Figure 8A and B. It was found that pretreatment of BDNF-shRNA notably blocked the increasing effects of 10 mg/kg cucurbitacin B on the amount of DCX^+^ cells in the DG of mice subjected to CUMS (1-way ANOVA: F_(6,28)_ = 35.514, *P < *.01, n = 5). In contrast, the usage of control-shRNA induced no effects on mice behaviors, BDNF signaling, and neurogenesis.

Furthermore, K252a, a potent pharmacological inhibitor of TrkB, was also used to block the BDNF system (Jiang et al., 2012; Wang et al., 2017; Zhang et al., 2019). Here, the CUMS-exposed mice were co-injected with cucurbitacin B (10 mg/kg) and K252a (25 μg/kg) for 2 weeks, and then the behavioral tests were performed (supplementary Figure 1A). As shown in supplementary Figure 1B–D, co-treatment with K252a and cucurbitacin B evidently attenuated the antidepressant-like effects of cucurbitacin B on mice exposed to CUMS in the FST (1-way ANOVA: F_(4,45)_ = 18.037, *P < *.01), TST (1-way ANOVA: F_(4,45)_ = 16.657, *P < *.01), and SPT (1-way ANOVA: F_(4,45)_ = 23.575, *P < *.01) in parallel with the BDNF-shRNA results in Figure 6B–D (n = 10). In summary, promotion of the hippocampal BDNF-TrkB system is required for the antidepressant-like effects of cucurbitacin B in mice.

### Blockade of Serotonin System Did Not Influence Antidepressant-Like Effects of Cucurbitacin B

Considering other hypotheses for the pathophysiology of depression, especially the monoamine hypothesis, we wanted to test whether the antidepressant-like effects of cucurbitacin B also require the serotonin system, and therefore, the tryptophan hydroxylase inhibitor PCPA was used (Jiang et al., 2012; Wang et al., 2017). Here, the CUMS-exposed mice were co-injected with cucurbitacin B (10 mg/kg) and PCPA (300 mg/kg) for 2 weeks, and then the behavioral tests were performed (supplementary Figure 2A). As shown in supplementary Figure 2B and C, mice in the (CUMS + cucurbitacin B + PCPA)-treated group showed similar immobility in the FST (1-way ANOVA: F_(4,45)_ = 21.423, *P < *.01) and TST (1-way ANOVA: F_(4,45)_ = 19.334, *P < *.01) to mice in the (CUMS + cucurbitacin B)-treated group (n = 10). Also, supplementary Figure 2D indicates that mice in the (CUMS + cucurbitacin B + PCPA)-treated group displayed nearly the same sucrose preference as mice in the (CUMS + cucurbitacin B)-treated group (1-way ANOVA: F_(4,45)_ = 27.023, *P < *.01, n = 10). In conclusion, the serotonin system does not participate in the antidepressant mechanism of cucurbitacin B.

## DISCUSSION

In recent years, the development of new antidepressants from natural herbal medicine has become one of the important research topics. Cucurbitacin B belongs to cucurbitacins, which are widely distributed and mainly isolated from plants in the Cucurbitaceae family (Dai et al., 2023). To date, the known cucurbitacins consist of cucurbitacin A-T, which can be divided into approximately 12 groups and has more than 200 derivatives (Cai et al., 2015). Among these compounds, cucurbitacin B, D, E, and I have been widely studied because of their strong anti-cancer activity (Chen et al., 2005). Furthermore, because cucurbitacin B is the most abundant and active form of cucurbitacin, it has received more attention from researchers than cucurbitacin D, E, and I (Dai et al., 2023). At present, research on the antitumor activity of cucurbitacin B is more extensive than that on its other pharmacological activities, and the reviews that can be retrieved from databases mainly refer to its antitumor effect (Dai et al., 2023). To our knowledge, the present study is the first comprehensive study showing that administration of cucurbitacin B not only produces significant antidepressant-like effects in the CUMS model of depression but also reverses the CUMS-induced dysfunction in the hippocampal BDNF signaling cascade and neurogenesis. Our findings extend the knowledge of the pharmacological activities of cucurbitacin B and may provide a novel antidepressant candidate.

In general, the main rationale for considering that cucurbitacin B may possess antidepressant-like efficacy derives from a previous report that demonstrated that cucurbitacin B treatment not only promoted hippocampal neurogenesis in mice but also induced the phosphorylation of 2 BDNF downstream molecules (ERK and CREB) in PC12 cells (Li et al., 2019) as a variety of compounds (P7C3, 7,8-dihydroxyflavone, etc.) with pro-neurogenic or pro-neurotrophic actions have been reported to possess antidepressant-like efficacy (Liu et al., 2010; Walker et al., 2015; Chang et al., 2016). Thus, both the FST and TST were used to initially evaluate cucurbitacin B, because they are widely adopted to screen potential antidepressant candidates (Cryan and Holmes, 2005; Cryan and Slattery, 2007). Our results were as expected. However, there may be a false-positive conclusion if cucurbitacin B has promoting actions on the locomotor activity of rodents, and therefore, the OFT was further used to evaluate cucurbitacin B. It was found that administration of cucurbitacin B did not influence mouse locomotor activity. Collectively, cucurbitacin B indeed has the potential to be an antidepressant candidate, and moreover, its usage may avoid the locomotor-related side effects found for some monoaminergic antidepressants in clinical practice. Some previous studies have suggested that the FST and TST could not evaluate “desperate state” or depressive-like behaviors but refer more to coping behavior or learning in animals (Molendijk and de Kloet, 2015; de Kloet and Molendijk, 2016). More reliable and convincing methods should also be used. It is well-known and widely accepted that CUMS models a chronic depressive-like state that gradually develops over time in response to various stresses and is considered to provide more natural induction (Willner, 1984, 1995; Forbes et al., 1996). CUMS induces behavioral changes in rodents that resemble clinical depression in human such as anhedonia and helplessness, while these behavioral parameters could be reversed by repeated administration of antidepressants used in clinical practice such as fluoxetine (Marona-Lewicka and Nichols, 1997; Yalcin et al., 2008; Mutlu et al., 2012). In addition to behavioral changes, CUMS also causes long-lasting changes of neurotrophic and neurogenic variables, and therefore, it is an excellent model for researching depression and evaluating antidepressants. Our finding that fluoxetine treatment significantly prevented the CUMS-induced depressive-like behaviors in mice helps support the effectiveness and reliability of our model. Similar to fluoxetine, cucurbitacin B treatment also produced beneficial effects against CUMS, suggesting that this compound may confer novel medications for the treatment of depression in the future.

Our western blotting and immunofluorescence data showed that the usage of cucurbitacin B fully ameliorated the inhibitory effects of CUMS on the hippocampal BDNF-TrkB system and neurogenesis in mice, consistent with Li et al. (Li et al., 2019). However, it was found that although cucurbitacin B treatment significantly increased the hippocampal BDNF expression and neurogenesis under depressive-like conditions, its administration did not achieve similar efficacy under normal conditions. For this phenomenon, currently we have no reasonable explanations. It is possible that for BDNF biosynthesis and neurogenesis in rodents, there are some negative feedback biological mechanisms that work under normal conditions but collapse under depressive-like conditions, similar to biological regulation of the hypothalamic-pituitary-adrenal axis activity (Juruena et al., 2018). Another confusing phenomenon is that although cucurbitacin B treatment did not affect the hippocampal BDNF expression in naïve control mice, its administration fully downregulated the immobility duration of them in the FST and TST. Here we speculate that under normal conditions, cucurbitacin B treatment may promote the biological activity but not expression of the hippocampal BDNF signaling cascade in rodents. Further in-depth molecular studies are ongoing in our groups. In this study, we studied only the hippocampus region. It is known that BDNF in some other regions than the hippocampus (medial prefrontal cortex, nucleus accumbens, etc.) is also implicated in the pathophysiology of depression (Xu et al., 2018; Koo et al., 2019). And so, BDNF in the medial prefrontal cortex and/or nucleus accumbens may also contribute to the antidepressant-like effects of cucurbitacin B in mice. To exclude this possibility, we adopted a strategy involving genetic knockdown of hippocampal BDNF in mice. The behavioral, western blotting, and immunofluorescence results involving BDNF-shRNA together confirm that the hippocampal BDNF-TrkB system was necessary for the antidepressant-like effects of cucurbitacin B in mice. Moreover, the behavioral results involving K252a and PCPA helped supporting this conclusion.

How does cucurbitacin B administration enhance the expression of hippocampal BDNF? We have noticed that in 2018, Kim et al. reported that cucurbitacin B induces a hypoglycemic effect in diabetic mice by promotion of AMP-activated protein kinase alpha (AMPKα) and glucagon-like peptide-1 via bitter taste receptor signaling (Kim et al., 2018). Previous studies have demonstrated that AMPKα activation leads to BDNF production in neurons (Yoon et al., 2008). Thus, it is possible that the enhanced effects of cucurbitacin B on BDNF biosynthesis were due to AMPKα activation. Moreover, BDNF is initially synthesized as a precursor protein (pro-BDNF). Pro-BDNF is converted to BDNF either intracellularly by furin/prohormone convertases or extracellularly by matrix metalloproteinase enzymes/plasmin (Qiao et al., 2017). Both pro-BDNF and BDNF are biologically active but have opposite functions (Qiao et al., 2017). It is also possible that cucurbitacin B has promoting effects on the conversion of pro-BDNF to BDNF. Both possibilities will be investigated in our further study.

Interestingly, a 2017 study demonstrated that cucurbitacin IIa, another cucurbitacin that belongs to category F, also exhibited significant antidepressant-like effects in the CUMS model (Zhou et al., 2017). Zhou et al. further showed that the antidepressant-like effects of cucurbitacin IIa may be exerted by promoting the calcium/calmodulin-dependent protein kinase II-CREB-BDNF pathway and regulating the balance between excitatory (involving GluN2B and GluA1) and inhibitory (involving GABA_A_-α2) synaptic transmission in the amygdala. As cucurbitacin B and cucurbitacin IIa have similar chemical structures, here we are wondering that the whole cucurbitacin family may be a valuable source for searching novel pro-neurogenic compounds and antidepressant candidates.

There may be a limitation for this study, as we have used only male C57BL/6J mice; female subjects were not included due to limited resources in our laboratory. Another shortcoming of this study was using only 1 rodent model of depression. In addition to CUMS, there are other acknowledged models of depression, such as chronic social defeat stress and chronic restraint stress models of depression. Our conclusion would be further confirmed if cucurbitacin B treatment also protects against chronic social defeat stress and chronic restraint stress. These limitations/shortcomings will be solved in the future. Moreover, this study did not include a control group in which BDNF-shRNA was given to nonstressed mice, also due to limited resources. A supplementary figure (Figure 13) in Jiang et al. has shown that the usage of BDNF-shRNA alone enhanced the immobility of mice in the FST and TST (Jiang et al., 2019). However, under the CUMS-induced depression-like conditions, the usage of BDNF-shRNA could not further enhance the immobility of mice exposed to CUMS, as we think there was a maximum limit for animal behaviors in the 2 tests. Regardless, the present study provides a new insight into understanding the pharmacological effects of cucurbitacin B and sheds light on the development of novel antidepressants with higher efficacy and fewer side effects.

## Supplementary Material

pyad052_suppl_Supplementary_Data

pyad052_suppl_Supplementary_Figure_S1

pyad052_suppl_Supplementary_Figure_S2

## Data Availability

The authors declare that all data supporting the findings of this study are available within the paper files.
