# Supplementary material for: Cucurbitacin B Exerts Significant Antidepressant-Like Effects in a Chronic Unpredictable Mild Stress Model of Depression: Involvement of the Hippocampal BDNF-TrkB System
Source: Int J Neuropsychopharmacol. 2023 Aug 21;26(10):680–91. doi: 10.1093/ijnp/pyad052 (PMC10586053; doi:10.1093/ijnp/pyad052)
Supplement: pyad052_suppl_Supplementary_Data [file pyad052_suppl_supplementary_data.docx]

**Supplemental Information**

**Supplemental Materials and Methods**

**Tail Suspension Test (TST)**

This test is also widely adopted to screen potential antidepressants. In the present study, the TST was performed according to Steru *et al*. with slight modifications (Steru et al., 1985). In brief, each test mouse was suspended from the top of the apparatus with their tail hung up on a rail by placing adhesive tape (approximately 1 cm from the tail tip). The immobility time was recorded during the last 4 min of the total 6-minute suspension (immobility means that mice stop struggling and stay still). Afterwards, each mouse was returned to its home cage. The observer was unaware of animal grouping.

**Open Field Test (OFT)**

Enhanced locomotor activity in rodents may contribute to reduction of immobility duration in the FST and TST, leading to false-positive conclusion (Bourin et al., 2001). To exclude this possibility, the OFT was adopted. In the present study, this test was performed according to Covington *et al*. with slight modifications (Covington et al., 2009). In brief, each test mouse was placed in the dark in an open field apparatus (100 × 100 × 40 cm) with the floor divided into 25 (5 × 5) squares. The apparatus was illuminated with a red bulb (50 W) on the ceiling. The squares each mouse crossed were counted over a 5-min period under dim light conditions. The open field apparatus was thoroughly cleaned after each trial. The observer was unaware of animal grouping.

**Chronic Unpredictable Mild Stress (CUMS)**

CUMS was the most widely acknowledged and used model of depression worldwide. Here, it was performed according to previous reports with slight modifications (Jiang et al., 2012; Ren et al., 2017; Zhang et al., 2019; Wu et al., 2022). During this experiment, the mice in the normal group were left undisturbed in the home cages in a separate room with exception of general handing (e.g., regular cage cleaning) that was matched by that for the CUMS groups. Mice were subjected once a day for 8 weeks to one of the following stressors: food or water deprivation for 24 h, cage tilting (45°C) for 24 h, damp bedding (200-ml water in 100-g sawdust bedding) for 24 h, tail clamp (1 cm from the beginning of the tail) for 1 min, swimming in cold water (4°C) for 5 min, and light/dark perversion for 24 h. To prevent habituation and provide an unpredictable feature to the stressors, all the stressors were randomly scheduled over a week and repeated throughout the 8-week period. Administration of cucurbitacin B/fluoxetine/K252a/PCPA/vehicle was performed daily in the last 2 weeks. To assess the depressive-like behaviors of mice, the FST, TST, and sucrose preference test (SPT) were performed together.

**SPT**

This test lasted for 4 days and was performed according to Pothion *et al*. with some modifications (Pothion et al., 2004). In brief, each mouse was given the choice to drink from two bottles in individual cages, one with 1% sucrose solution and the other with water. All mice were acclimatized to the two-bottle choice condition for 2 days, and the position of the two bottles was changed every 6 h to prevent potential location preference of drinking. Then, all mice were deprived of food and water for 18 h prior to the test. On each test, the two bottles were pre-weighed and the position was interchanged. After 6 h, the amount of sucrose solution or water consumed was determined by weighing the bottles again. Sucrose preference (%) = consumption × 100/(sucrose consumption + water consumption). The observers were unaware of animal grouping.

**Genetic Knockdown of Hippocampal BDNF**

AAV-BDNF-shRNA-EGFP and AAV-Control-shRNA-EGFP used in the present study were provided by GeneChem, and their production has been described in previous reports (Liu et al., 2020; Huang et al., 2023). Here, each mouse anesthetized with 0.5% pentobarbital sodium was fixed in a stereotactic frame (Stoelting, Wood Dale, USA). After cutting the scalp, the skull of each mouse was exposed using 75% ethanol and 1% H_2_O_2_. Two small drill holes were bilaterally made on the skull, and then, a 10 μl Hamilton syringe was placed at the hippocampus coordinates: AP = − 2.3 mm, ML = ± 1.5 mm, DV = + 1.4 mm (CA1) and 1.8 mm (DG) (Wang et al., 2017; Zhang et al., 2019). AAV-BDNF-shRNA or AAV-Control-shRNA was bilaterally infused into the hippocampus region of each mouse using the syringe at a rate of 0.5 μl/min (1.5 µl/side: 1 µl for CA1, 0.5 µl for DG). Afterwards, 5 min of waiting was needed to prevent AAV reflux. The wound of each mouse was cleaned and sutured. A period of 2 weeks was required for the expression of AAV to be stable in the hippocampus. Furthermore, these animals were subjected to 8 weeks of CUMS and 2 weeks administration of cucurbitacin B/vehicle, followed by the FST, TST, and SPT.

All AAVs were adjusted to 1 × 10^12^ TU/ml before use. The nucleotide sequences for BDNF-shRNA and Control-shRNA were 5’-TGAGCGTGTGTGACAGTATTA-3’ and 5’-TTCTCCGAACGTGTCACGT-3’, respectively (Liu et al., 2020; Huang et al., 2023).

**Western Blotting**

This method was performed according to many previous reports (Ren et al., 2017; Wang et al., 2017; Zhang et al., 2019; Liu et al., 2020; Wu et al., 2022; Huang et al., 2023). After sacrifice, the hippocampus tissues were immediately dissected from each mouse and stored at -80°C. Protein samples were extracted from the tissues using NP-40 lysis buffer. After quantification and denaturation, 30 μg of protein samples were loaded and separated by 10/12% SDS/PAGE gels and then transferred to nitrocellulose membranes. After proteins blocking and TBST washing, membranes were incubated overnight at 4°C with primary antibodies against BDNF (1:500; ab108319; Abcam, Bristol, UK), TrkB (1:1000; ab187041; Abcam), p-TrkB-Tyr516 (pTrkB; 1:500; PA5-36695; Thermo Fisher, Waltham, USA), ERK1/2 (1:500; 4695S; Cell signaling, Danvers, USA), p-ERK1/2-Thr202/Tyr204 (pERK1/2; 1:500; 4370S; Cell Signaling), AKT (1:500; 9272S; Cell Signaling), p-AKT-Ser473 (1:500; 9271S; Cell Signaling), CREB (1:500; 9197S; Cell Signaling), p-CREB-Ser133 (1:500; 9198S; Cell Signaling), and β-actin (1:5000; 4967S; Cell Signaling). After TBST washing again, membranes were incubated with IR-Dye 680-labelled secondary antibodies (1:10000) for 2 h at room temperature. An Odyssey CLx system (Licor, Lincoln, USA) was used to detect the bands.

**Immunofluorescence**

In the present study, the level of hippocampal neurogenesis in mice was determined by doublecortin (DCX) immunofluorescence. The procedures were performed according to previous studies (Liu et al., 2020; Huang et al., 2023). In brief, mice anesthetized with 0.5% sodium pentobarbital were subjected to transcardial perfusion of 4% paraformaldehyde. After post-fixation and dehydration, 25 μm of hippocampal slices were collected. For DCX staining, the slices were dealt in a commonly adopted manner: 1, 0.3% Triton X-100 incubation; 2, 3% BSA incubation; 3, primary antibody incubation; 4, washed in PBS; 5, secondary antibody incubation; 6, washed in PBS; 7, 4’,6-diamidino-2-phenylindole (DAPI) incubation; 8, washed in PBS; 9, cover-slipped and observed. Primary antibody against DCX (1:100; 4604S; Cell signaling) and fluorescein isothiocyanate (FITC)-labeled secondary antibody (1:50; Thermo Fisher) were used. The method of DCX examination has also been described as before (Huang et al., 2023). A FV1000 confocal fluorescence microscopy (Olympus, Tokyo, Japan) was used to observe the slices. Examinations of the DCX-labeled (DCX^+^) cells were confined to the DG sub-region in the hippocampus, especially in granule cell layer (GCL) and also sub-granular zone (SGZ) which was defined as a two-cell body wide zone along the border between GCL and hilus. Quantifications of the DCX^+^ cells were respectively conducted from 1-in-6 series of the hippocampal sections spaced at 150 μm and spanning the rostrocaudal region (AP from -1.22 mm to -2.80 mm) of DG bilaterally. Every DCX^+^ cell within GCL and SGZ was counted.

**References**

Bourin M, Fiocco AJ, Clenet F (2001) How valuable are animal models in defining antidepressant activity? *Hum Psychopharmacol* **16**:9-21.

Covington HE, 3rd, Maze I, LaPlant QC, Vialou VF, Ohnishi YN, Berton O, Fass DM, Renthal W, Rush AJ 3rd, Wu EY, Ghose S, Krishnan V, Russo SJ, Tamminga C, Haggarty SJ, Nestler EJ (2009) Antidepressant actions of histone deacetylase inhibitors. *J Neurosci* **29**:11451-11460.

Huang J, Fan H, Chen YM, Wang CN, Guan W, Li WY, Shi TS, Chen WJ, Zhu BL, Liu JF, Jiang B (2023) The salt-inducible kinases inhibitor HG-9-91-01 exhibits antidepressant-like actions in mice exposed to chronic unpredictable mild stress. *Neuropharmacology* **227**:109437.

Jiang B, Xiong Z, Yang J, Wang W, Wang Y, Hu ZL, Wang F, Chen JG (2012) Antidepressant-like effects of ginsenoside Rg1 are due to activation of the BDNF signalling pathway and neurogenesis in the hippocampus. *Br J Pharmacol* **166**:1872-1887.

Liu Y, Tang W, Ji C, Gu J, Chen Y, Huang J, Zhao X, Sun Y, Wang C, Guan W, Liu J, Jiang B (2020) The Selective SIK2 Inhibitor ARN-3236 Produces Strong Antidepressant-Like Efficacy in Mice via the Hippocampal CRTC1-CREB-BDNF Pathway. *Front Pharmacol* **11**:624429.

Pothion S, Bizot JC, Trovero F, Belzung C (2004) Strain differences in sucrose preference and in the consequences of unpredictable chronic mild stress. *Behav Brain Res* **155**:135-146.

Ren Y, Wang JL, Zhang X, Wang H, Ye Y, Song L, Wang YJ, Tu MJ, Wang WW, Yang L, Jiang B (2017) Antidepressant-like effects of ginsenoside Rg2 in a chronic mild stress model of depression. *Brain Res Bull* **134**:211-219.

Steru L, Chermat R, Thierry B, Simon P (1985) The tail suspension test: a new method for screening antidepressants in mice. *Psychopharmacology (Berl)* **85**:367-370.

Wang H, Zhao Y, Wang YJ, Song L, Wang JL, Huang C, Zhang W, Jiang B (2017) Antidepressant-like effects of tetrahydroxystilbene glucoside in mice: Involvement of BDNF signaling cascade in the hippocampus. *CNS Neurosci Ther* **23**:627-636.

Wu ZH, Fan H, Gao SY, Jin YF, Cheng C, Jiang B, Shen J (2022) Antidepressant-like activity of oroxylin A in mice models of depression: A behavioral and neurobiological characterization. *Front Pharmacol* **13**:921553.

Zhang JJ, Gao TT, Wang Y, Wang JL, Guan W, Wang YJ, Wang CN, Liu JF, Jiang B (2019) Andrographolide Exerts Significant Antidepressant-Like Effects Involving the Hippocampal BDNF System in Mice. *Int J Neuropsychopharmacol* **22**:585-600.

**Supplemental Figure Legends**

Figure S1. Pharmacological blockade of the hippocampal BDNF-TrkB system significantly attenuated the antidepressant-like effects of cucurbitacin B on mice behaviors. (A) A schematic diagram which describes the timeline of the experimental procedures. Mice exposed to CUMS (8 weeks) were given co-administration of cucurbitacin B (10 mg/kg) and K252a (25 μg/kg) daily during the last 2 weeks, followed by the FST, TST, and SPT. (B and C) Mice in the (CUMS + Cucurbitacin B + K252a)-treated group displayed significantly more immobility in both the FST and TST than mice in the (CUMS + Cucurbitacin B)-treated group. (D) Mice in the (CUMS + Cucurbitacin B + K252a)-treated group had notably less sucrose preference in the SPT than mice in the (CUMS + Cucurbitacin B)-treated group. All results are expressed as means ± S.E.M. (n = 10); ^**^*P*< 0.01 vs. Vehicle; ^##^*P*< 0.01 vs. (CUMS + Vehicle). For statistical analyses, one-way ANOVA and Tukey’s test were used together.

Figure S2. Blockade of the serotonin system did not influence the antidepressant-like effects of cucurbitacin B in mice. (A) A schematic diagram which describes the timeline of the experimental procedures. Mice exposed to CUMS (8 weeks) were given co-administration of cucurbitacin B (10 mg/kg) and PCPA (300 mg/kg) daily during the last 2 weeks, followed by the FST, TST, and SPT. (B and C) Mice in the (CUMS + Cucurbitacin B + PCPA)-treated group displayed similar level of immobility in both the FST and TST than mice in the (CUMS + Cucurbitacin B)-treated group. (D) Mice in the (CUMS + Cucurbitacin B + PCPA)-treated group showed similar level of sucrose preference in the SPT than mice in the (CUMS + Cucurbitacin B)-treated group. All results are expressed as means ± S.E.M. (n = 10); ^**^*P*< 0.01 vs. Vehicle; ^##^*P*< 0.01 vs. (CUMS + Vehicle). For statistical analyses, one-way ANOVA and Tukey’s test were used together.
