# Supplementary figures and images for: Cucurbitacin B Exerts Significant Antidepressant-Like Effects in a Chronic Unpredictable Mild Stress Model of Depression: Involvement of the Hippocampal BDNF-TrkB System
Source: Int J Neuropsychopharmacol. 2023 Aug 21;26(10):680–91. doi: 10.1093/ijnp/pyad052 (PMC10586053; doi:10.1093/ijnp/pyad052)

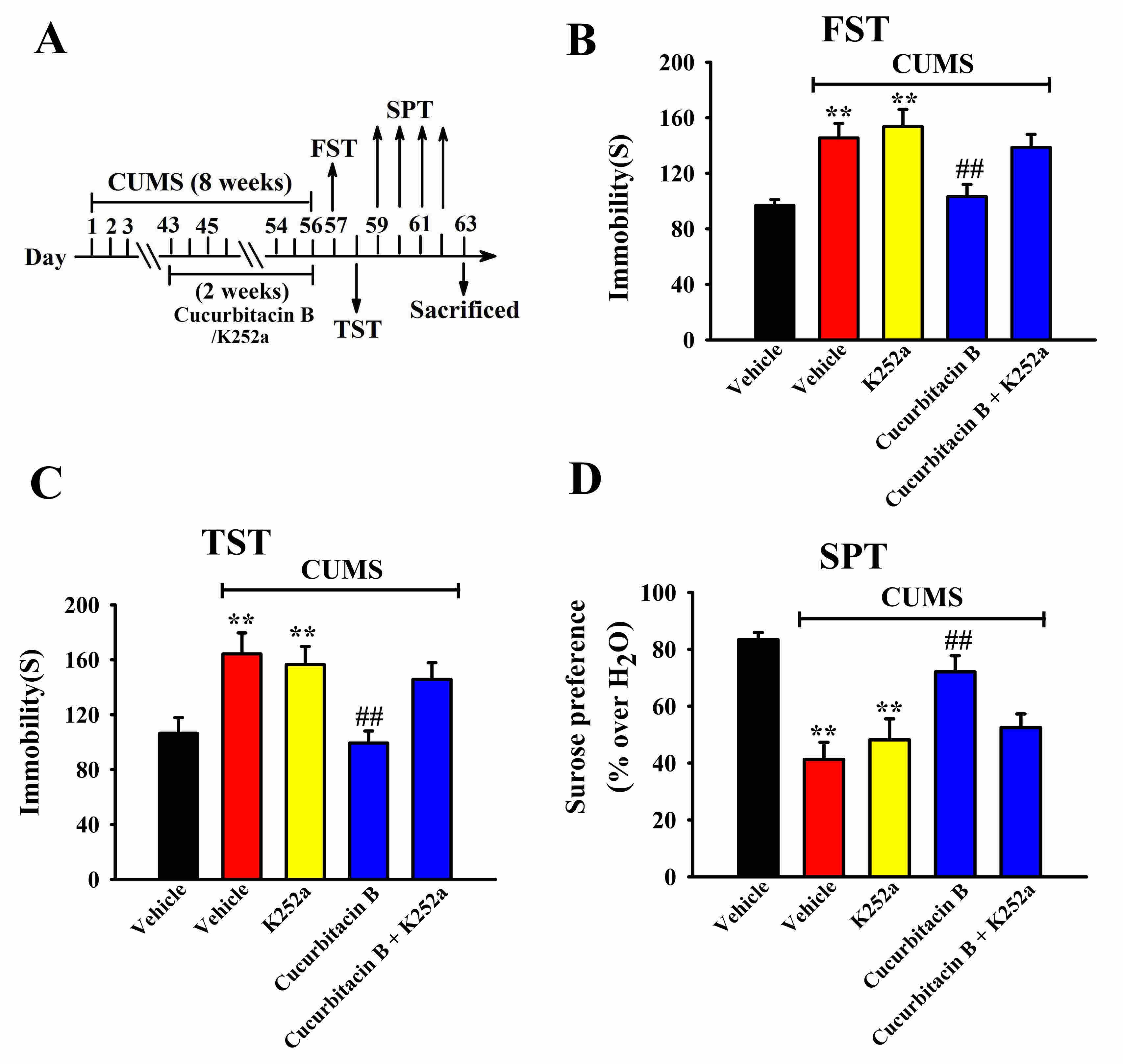

Supplement: pyad052_suppl_Supplementary_Figure_S1 [file pyad052_suppl_supplementary_figure_s1.jpeg]

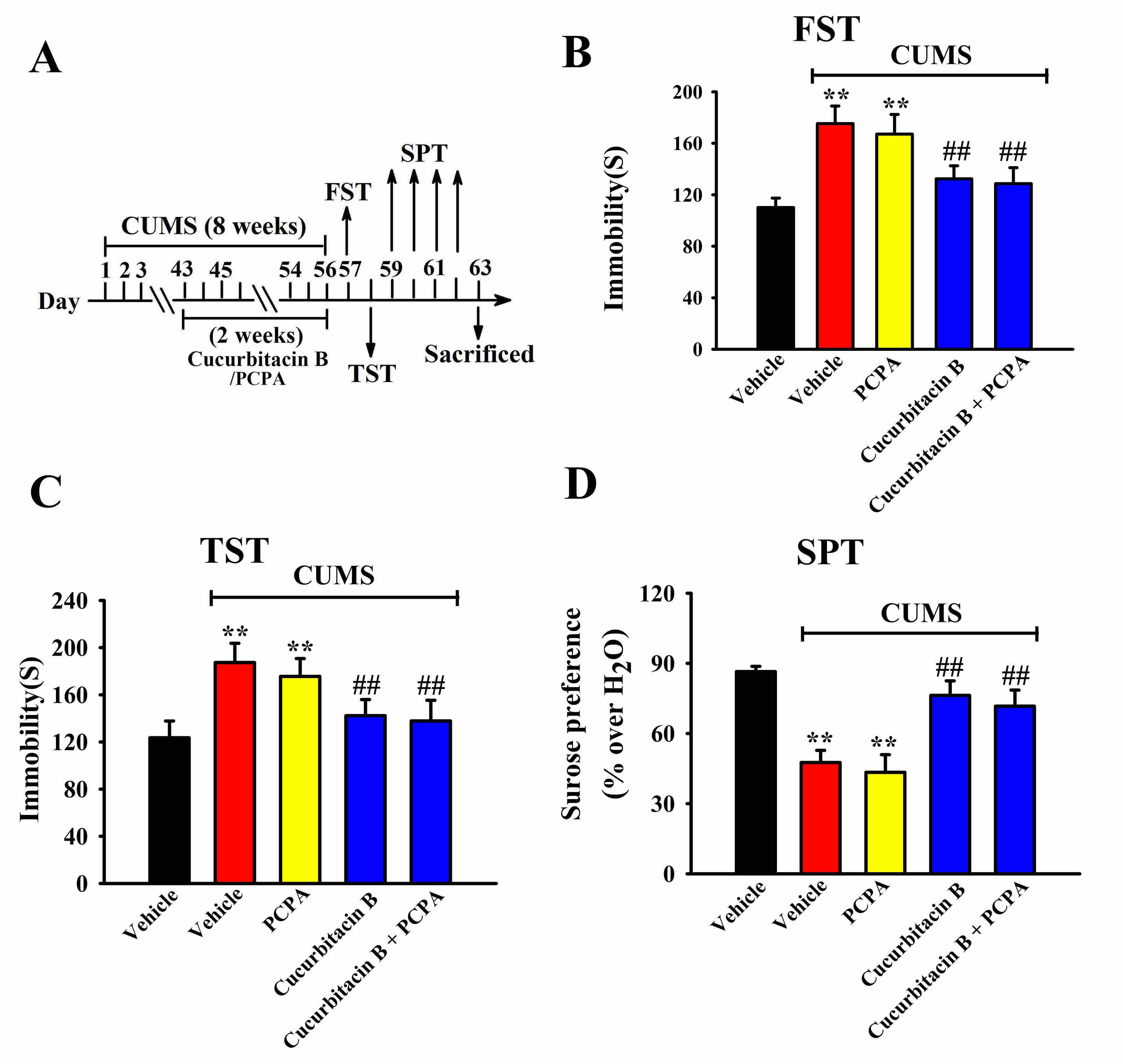

Supplement: pyad052_suppl_Supplementary_Figure_S2 [file pyad052_suppl_supplementary_figure_s2.jpeg]
